# *Eubacterium rectale* Attenuates HSV-1 Induced Systemic Inflammation in Mice by Inhibiting CD83

**DOI:** 10.3389/fimmu.2021.712312

**Published:** 2021-08-31

**Authors:** S. M. Shamsul Islam, Hye-Myung Ryu, Hasan M. Sayeed, Hae-Ok Byun, Ju-Yang Jung, Hyoun-Ah Kim, Chang-Hee Suh, Seonghyang Sohn

**Affiliations:** ^1^Department of Biomedical Science, Ajou University School of Medicine, Suwon, South Korea; ^2^Department of Microbiology, Ajou University School of Medicine, Suwon, South Korea; ^3^Department of Rheumatology, Ajou University School of Medicine, Suwon, South Korea; ^4^Department of Molecular Science and Technology, Ajou University, Suwon, South Korea

**Keywords:** Behçet’s disease, *Eubacterium rectale*, dendritic cells, microbiota, inflammation, CD83

## Abstract

The purpose of this study was to determine whether administration of the microorganism *Eubacterium rectale* (*E. rectale*) could regulate dendritic cell (DC) activation and systemic inflammation in herpes simplex virus type 1-induced Behçet’s disease (BD). *E. rectale*, butyrate-producing bacteria, was administered to BD mice. Peripheral blood leukocytes (PBL) and lymph node cells were isolated and analyzed by flow cytometry. 16S rRNA metagenomic analysis was performed in the feces of mice to determine the differences in the composition of the microbial population between normal and BD mice. Serum cytokine levels were measured by enzyme-linked immunosorbent assay. The frequency of DC activation marker CD83 positive cells was significantly increased in PBL of BD mice. Frequencies of CD83+ cells were also significantly increased in patients with active BD. 16S rRNA metagenomic analysis revealed different gut microbiota composition between normal and BD mice. The administration of *E. rectale* to BD mice reduced the frequency of CD83+ cells and significantly increased the frequency of NK1.1+ cells with the improvement of symptoms. The co-administration of colchicine and *E. rectale* also significantly reduced the frequency of CD83+ cells. Differences in gut microbiota were observed between normal mice and BD mice, and the administration of *E. rectale* downregulated the frequency of CD83, which was associated with BD deterioration. These data indicate that *E. rectale* could be a new therapeutic adjuvant for BD management.

## Introduction

The exact cause of Behçet’s disease (BD) is unknown (1, 2). BD is characterized by chronic systemic inflammation accompanied by oral aphthous ulcers, genital ulcers, skin lesions, ocular involvement, arthritis, and gastrointestinal and central nervous system involvement (1, 2). Herpes simplex virus (HSV) is considered one of the causative factors of BD pathogenesis, and HSV viral DNA particles have been detected in the ocular fluids (3), peripheral blood (4), saliva (5), and skin lesions (6) of BD patients. BD has been reported to be associated with significant gut microbiota changes (6). The gut provides a habitat for microbes, and microbes in the gut microenvironment have been shown to influence many physiological conditions (7). The microbiota has been described as contributing to immune system balance to maintain host homeostasis (8) and understanding the normal gut microbiota has made the therapeutic manipulation of the gut ecosystem a rational and realistic future prospect (9). Alterations in the microbial community can impair immune regulation, which can lead to autoimmune disorders (10). BD is a T-helper cell type 1 (Th1) polarized disease (11) and is a chronic inflammatory disease associated with significant gut microbiome changes (12).

HSV-1 induced BD model mice show clinical symptoms similar to those of BD patients, including genital ulcers, oral ulcers, skin lesions, eye involvement, arthritis, and intestinal involvement (13). Although the pathogenesis of BD is not completely clear, genetic susceptibility, triggering factors, and immunological abnormalities have been reported to play roles in BD development (1). BD has also been reported to result from T cell abnormalities, Th1/Th2 imbalance, Th17 polarization (14), and the involvement of the innate immune system (15–17). Organ-specific autoimmune diseases are mediated by an imbalance of T cell subsets, and autoreactive T cells are activated by dendritic cells (DC) (18). Dendritic cells are known as the most potent antigen-presenting cells and are essential for T cell activation. The activation of T cells is essential for a successful cellular immune response (19). To activate naive T cells, T cells require at least two signals from DC. The first signal is antigen specificity, which passes through the T cell receptor (TCR) through the major histocompatibility complex (MHC), causing T cells to enter the cell cycle. Upon peptide accumulation, the second signal is activated where DC expand the level of expression of co-stimulatory molecules such as CD40, CD83, CD80, and CD86 for T cell proliferation and cytokine production (19–22). Among the co-stimulatory molecules, CD83 plays an important role in the immune response beyond its function as an activation marker (23). CD83 is a member of the Ig gene superfamily and an important marker for the characterization of mature DCs but can be detected on other immune cells (24, 25). The downregulation of CD83 through RNA interference in human DC results in the less potent induction of allogeneic T cell proliferation and decreases interferon (IFN)-γ secretion (21). CD83 inhibition by CD83 siRNA was reported to improve the symptoms of BD mice (26).

The production of butyrate by gut microbiota is usually associated with a healthy intestinal environment. Active BD patients showed decreased butyrate production (27). *Eubacterium rectale* (*E. rectale*), which accounts for up to 13% of the gut microbiota in total feces in the human colon and thus, is one of the most prevalent bacterial species, is a major contributor to the production of butyrate (28). Patients with Crohn’s disease (29, 30), rheumatoid arthritis (RA) (31), and ulcerative colitis (32) have significantly reduced amounts of *E. rectale*, and these patients have lower butyrate concentrations in their feces than healthy individuals. Butyrate treatment downregulated IL-6 in peripheral blood mononuclear cell cultures of patients with BD (33) and the downregulation of IL-6 was associated with BD symptom improvement in mice (34). Metagenomics studies have shown that the gut microbiota is associated with human diseases (35). Therefore, the future therapeutic potential of the gut microbiota remains to be further demonstrated (36). In BD, impaired microbiota may contribute to the development of symptoms (27). However, there is little evidence that specific microbial regulation is directly related to improving symptoms. In this study, we investigated the microbiota associated with BD in the HSV-1 induced BD mouse model and demonstrated that *E. rectale* regulated DC activation, leading to an improvement in BD.

## Materials and Methods

### Patients

Patients with BD were diagnosed according to the International Study Group of Behçet’s Disease (37). The BD activity index was calculated as outlined in the BD Current Activity Form 2006 (http://medhealth.leeds.ac.uk/download/910/behcetsdiseaseactivityform). Disease was evaluated following a consultant-led assessment at the time of sample collection. The patients were defined as having active BD (BDA) if they presented with two or more of the following disease manifestations: oral aphthous ulcers; genital ulcers; positive pathergy test; skin lesions; ocular, vascular, and neurological involvement; and arthritis. Blood sampling was done at the active stage and the inactive stage (BDI) during follow-up after improvement of the symptoms. The patients were recruited from the Department of Rheumatology at Ajou University Hospital, Republic of Korea. The medical history and the clinical characteristics of all subjects were collected by a review of the medical records and an interview with the subject when the samples were collected. This study was approved by the Institutional Review Board of Ajou University Hospital. All subjects provided signed informed consent (approval number: AJIRB-BMR-SMP-13-398).

### Animal Experiment

Institute of Cancer Research (ICR) mice 4 to 5-weeks-old were infected with HSV type 1 (1x10^6^ pfu/mL, F strain) grown in Vero cells as previously described (13). Virus inoculation was performed twice at 10-day intervals followed by 16 weeks of observation. The mice were bred in temperature- and light-controlled conventional rooms (20 - 22°C, 12 h light/dark cycle). The mice had free access to food and water. During the experimental period, the animals were closely observed and photographed. The mice were handled in accordance with the protocol approved by the Institutional Animal Care and Use Committee of Ajou University (approval number: AMC-2018-0017).

### BD Symptomatic Mice Induced by HSV-1

Virus inoculation was performed using published procedures (13). Briefly, the earlobes of mice were scratched with a needle and inoculated with 20 μL of 1 x 10^6^ pfu/mL HSV-1 (F strain) that had been grown in Vero cells. Virus inoculation was performed twice at 10-day intervals. For virus inoculation, the mice were anesthetized by intraperitoneal injection with 2,2,2-tribromo ethanol (240 mg/kg), inoculated with virus, and followed up for five to sixteen weeks to confirm BD symptoms. Several symptoms were observed in the mice following HSV inoculation. BD symptoms including oral ulcers, genital ulcers, erythema, skin pustules, skin ulcers, arthritis, diarrhea, red-eye, loss of balance, and facial swelling were observed in 10% of the HSV-inoculated mice. Oral, genital, skin ulcers, and eye symptoms were classified as major symptoms while arthritis, intestinal ulceration, and neurological involvement were considered minor symptoms. Mice with one or more major symptoms and one or more minor symptoms were classified as having BD. Each symptom was assigned a score of one. The sum of the different symptom scores was used to determine the severity of BD using the BD current activity form 2006 prepared by the International Society for Behçet’s Disease (http://medhealth.leeds.ac.uk/download/910/behcetsdiseaseactivityform). A loss of symptoms or a reduction in lesion size of more than 20% was an indicator of BD improvement. HSV-1 inoculated asymptomatic mice were used as BD normal (BDN) mice as previously described (13).

### Study Design

**Figure 1:**
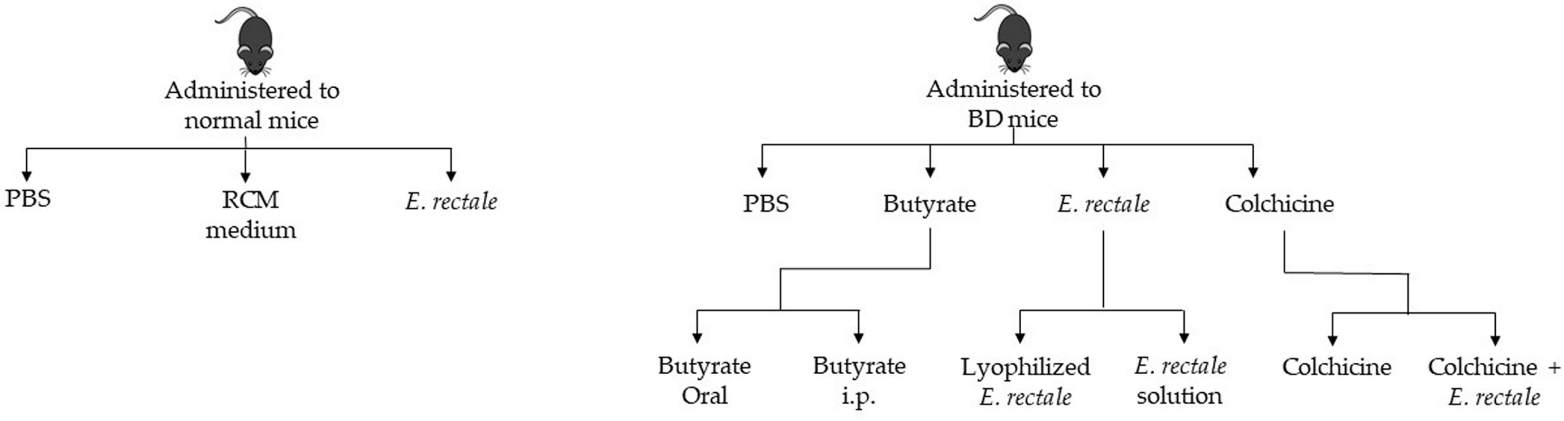
Schematic diagram of the *in vivo* study.

Normal healthy mice were randomly divided into three groups and administered 1. phosphate-buffered saline (PBS); 2. RCM medium; and 3. *E. rectale*. BD mice were randomly divided into four groups: 1. PBS-treated BD mice; 2. butyrate-treated BD mice; 3. *E. rectale*-treated BD mice; and 4. colchicine-treated BD mice. The butyrate-treated BD mice were randomly divided into two groups: 1. oral administration of butyrate and 2. intraperitoneal injection of butyrate. The *E. rectale*-treated BD mice were randomly grouped. Group 1 was BD mice treated with *E. rectale* in culture media and Group 2 was freeze-dried *E. rectale*. Colchicine-treated BD mice were randomized into two different groups: colchicine-treatment and colchicine with *E. rectale* treatment (**Figure 1**).

### 16S rRNA Metagenomic Analysis

To analyze the mouse gut microbiota based on 16S rRNA gene amplicon sequences, fresh feces were collected from normal and BD symptomatic mice. 16S rRNA gene sequencing of the V3 and V4 amplicons was performed using 16S rRNA gene PCR primer (forward primer 5′-TCG TCG GCA GCG TCA GAT GTG TAT AAG AGA CAG CCT ACG GGN GGC WGC AG-3′, reverse primer 5′-GTC TCG TGG GCT CGG AGA TGT GTA TAA GAG ACA GGA CTA CHV GGG TAT CTA ATC C-3′). The number of operational taxonomic units (OTUs) was determined by clustering the sequence from each sample with 97% sequence identity as a cutoff using quantitative insights into microbial ecology (QIIME) software (v.1.8.0). Taxonomic abundance was counted with a National Center for Biotechnology Information (NCBI) database using a confidence threshold of 0.8 derived from preprocessed reads for each sample.

### Bacteria Culture

The bacterial strain *E. rectale* KCTC 5835 was purchased from the Korean Collection for Type Cultures (KCTC). *E. rectale* was cultured anaerobically for five days at 37°C in enriched Clostridium medium (RCM) broth (Sparks Becton, MD) supplemented with 0.2 g/L L-cystine and 4 g/L Na_2_HPO_4_.

### Preparation of Lyophilized *E. rectale*

*E. rectale* was freeze-dried at -85°C for 24 hours using an Ilshin FDS8508 freeze-dryer (Gwacheon-si, Gyeonggi-do, Korea). After freeze-drying, the product was weighed, diluted in RCM, and administered orally to the mice.

### Medication Administration

After the onset of BD symptoms, mice were orally administered with 1.7x10^8^ or 1.0x10^9^ colony-forming unit (C.F.U) of *E. rectale* bacteria once a day for 10 consecutive days. As a control group, BD mice were administered RCM (*E. rectale* culture media) for the same period. In addition, butyrate was administered orally or intraperitoneally to BD mice for 10 days. Colchicine 2 ug per mouse was orally administered to BD mice once a day for 10 consecutive days. The combination of colchicine and *E. rectale* was also administered to BD mice during the same period.

### Flow Cytometric Analysis

Leukocytes were isolated from peripheral blood (PBL) and stained with anti-mouse CD40, CD83, CD80, and CD86 for 30 min at 4°C. In addition, cells isolated from peripheral blood and LN were stained with CD4, CD11c, CD8, CD11b, and NK1.1 and analyzed with a FACS Aria III flow cytometer (Becton Dickinson, San Jose, CA, USA). Sources of antibodies used in humans and mice are listed in (**Supplementary Table ST1**).

### Regulatory T Cell FACS Staining

To identify regulatory T (Treg) cells, isolated PBL were stained with anti-mouse CD4 and anti-mouse CD25. To detect intranuclear Foxp3, an anti-mouse Foxp3 staining kit (eBioscience, San Diego, CA, USA) was used according to the manufacturer’s instructions. Briefly, the cells were fixed using Fix/Perm buffer, washed with 1x permeation buffer, and incubated with anti-Foxp3 antibody. Then, more than 10,000 cells were analyzed by flow cytometry (FACS Aria III, Becton Dickinson, CA, USA).

### Measurement of Cytokine IL-17 by Enzyme-Linked-Immunosorbent Assay (ELISA)

IL-17 levels were analyzed in mouse plasma using a commercial ELISA kit (R&D Systems, Minneapolis, MN, USA) according to the manufacturer’s instructions. The absorbance values of the samples were read at a wavelength of 450 nm using a Bio-Rad model 170-6850 microplate reader (Hercules, CA, USA). ELISA was repeated in duplicate wells.

### Statistical Analysis

Statistical differences between the experimental groups were determined using Graphpad Prism for Windows (version 8.3.1) (GraphPad Software, La Jolla, CA, USA). The statistical significance of the data was determined by applying the Kruskal-Wallis test and the Mann-Whitney U test. The data are expressed as the mean ± SD and statistical significance was considered when the p-value was less than 0.05.

## Results

### Clinical Characteristics of the Patients

The clinical characteristics of the patients with BD are summarized in **Table 1**. Blood sampling was performed twice (active stage and the inactive stage of the disease). The second sampling was performed after symptom improvement. In active BD patients, oral ulcers were found in 8 patients (57.12%), genital ulcers in 2 patients (14.28%), arthritis in 12 patients (85.68%), gastrointestinal inflammation in 1 patient (7.14%), and nodular erythema in 4 patients (25.6%). Medication is shown in **Table 2**. Colchicine in 11 (78.6%), glucocorticoid in 9 (64.3%), azathioprine in 1 (7.14%), hydroxychloroquine in 3 (21.4%), sulfasalazine in 5 (35.7%), nonsteroidal anti-inflammatory drugs were administered to 9 patients (64.3%) of active BD patients.

**Table 1:** Clinical characteristics of the Behçet’s disease patients.

Patient	age	sex	OU	GU	Arthritis	GI	EN	Pathergy	HLA-B51
Active									
1	42	M	–	–	+	–	–	–	–
2	41	F	+	–	+	–	+	–	–
3	36	M	+	–	+	–	–	–	–
4	46	F	+	–	+	–	+	–	–
5	42	F	–	–	+	–	–	–	–
6	49	F	–	–	+	–	–	–	–
7	55	F	+	–	+	–	+	–	–
8	42	F	–	–	+	–	–	–	–
9	26	F	–	–	+	–	–	–	–
10	51	M	+	–	–	–	–	–	–
11	48	F	–	–	–	–	–	–	–
12	58	F	+	+	+	+	+	–	–
13	50	F	+	+	+	–	–	–	–
14	53	F	+	–	+	–	–	–	–
Inactive									
1	42	M	–	–	+	–	–	–	–
2	42	F	+	–	+	–	+	–	–
3	57	F	–	–	+	–	–	–	–
4	36	F	+	+	–	–	–	–	+
5	38	M	+	–	+	–	–	–	–
6	27	F	+	–	+	–	–	–	–
7	54	F	–	–	–	–	–	–	–

M, male; F, female; OU, oral ulcers; GU, genital ulcers; GI, gastrointestinal inflammation; EN, erythema nodosum.

**Table 2:** Therapeutic history of Behçet’s disease patients.

Patient	Colchicine	Steroid	AZP	Bucillamine	HCQ	minocycline	SZP	NSAIDs
Active								
1	+	+	+					
2	+	+					+	+
3	+						+	+
4	+						+	+
5	+	+					+	+
6	+	+			+			
7	+	+			+			+
8								+
9	+	+			+			+
10	+	+						
11								
12	+	+						+
13								
14	+	+					+	+
Inactive								
1	+	+	+					+
2	+				+		+	
3		+	+				+	+
4	+						+	
5	+						+	+
6	+				+			+
7	+	+						

AZP, azathioprine; HCQ, Hydroxychloroquine; SZP, Sulfasalazine; NSAIDs, Nonsteroidal anti-inflammatory drugs.

### Frequency of CD83-Expressing Cells in Healthy Control, Active BD, and Inactive BD Patients

**Figure 2:**
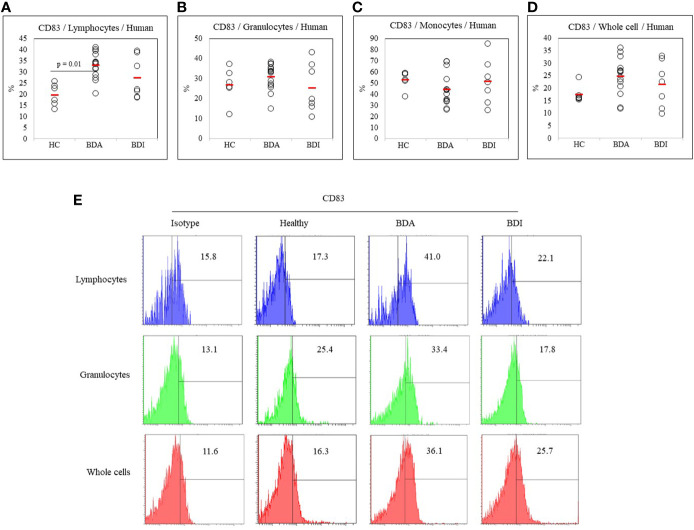
Frequency of CD83+ cells in peripheral blood leukocytes (PBL), lymphocytes, granulocytes, monocytes, and whole cells **(A–D)** of healthy controls (HC), patients with active Behçet’s disease (BDA), and patients with inactive Behçet’s disease (BDI). The isolated peripheral blood leukocytes (PBL) were subjected to surface staining and analyzed by flow cytometry. The results were obtained from six HC, fourteen BDA patients, and seven BDI patients. Statistical analysis was performed by the Kruskal-Wallis test. Representative FACS histograms of peripheral blood lymphocytes, granulocytes, and whole cells are shown in **(E)**.

The frequency of DC-activating co-stimulatory molecule CD83 in active BD patients (n = 14), inactive BD patients (n = 7), and healthy controls (n = 6) was determined by FACS analysis. The frequency of CD83+ cells were significantly increased in the lymphocytes (33.03 ± 5.67% *vs.* 19.75 ± 5.02%, p = 0.01) from BDA patients compared with the HC (**Figures 2A**). No significant difference was found in the frequency of CD83+ granulocyte, monocyte, and whole cell populations (**Figures 2B–D**). A representative histogram of CD83+ cells from the HC, BDA, and BDI groups is shown in **Figure 2E**.

### Frequency of CD83, CD86, CD40, and CD80-Expressing Cells in Normal Control, BD Normal (BDN), and BD Mice

**Figure 3:**
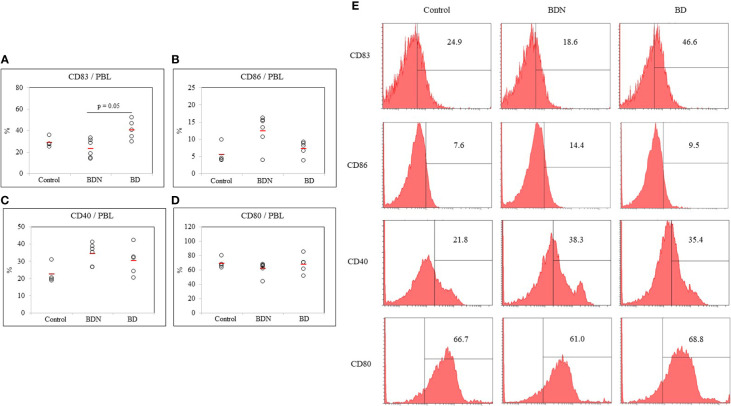
The frequency of DC costimulatory molecules CD83, CD86, CD40, and CD80 **(A–D)** in whole PBL was assessed in normal, BDN, and BD mice. Isolated PBL were surface stained and evaluated by flow cytometry. A representative histogram of CD83+ cells is shown in **(E)**. For statistical analysis, the Kruskal-Wallis test was performed using GraphPad. The number of mice used in the experimental groups was five in the normal, six in the BDN, and five in the BD group. The experiments were performed independently at least three times.

The frequency of DC expressing co-stimulatory molecules CD83, CD86, CD40, and CD80 in the PBL of mice were analyzed by FACS. The frequency of CD83+ cells in HSV-1 induced BD mice was significantly elevated compared to those in the BDN mice (40.72 ± 8.91% *vs.* 23.31 ± 8.51%, *p* = 0.05) (**Figure 3A**). The frequency of CD86+ cells was decreased in BD mice compared to BDN mice (7.28 ± 2.18% *vs.* 12.45 ± 4.62%) but was not statistically significant (**Figure 3B**). There was no statistically significant difference in the frequency of CD40+ and CD80+ expressing cells between groups (**Figures 3C, D**). **Figure 3E** shows representative histograms of CD83+, CD86+, CD40+, CD80+ cells in control, BDN, and BD mice.

### Altered Gut Microbial Composition in HSV-1-Induced BD Symptomatic Mice

**Figure 4:**
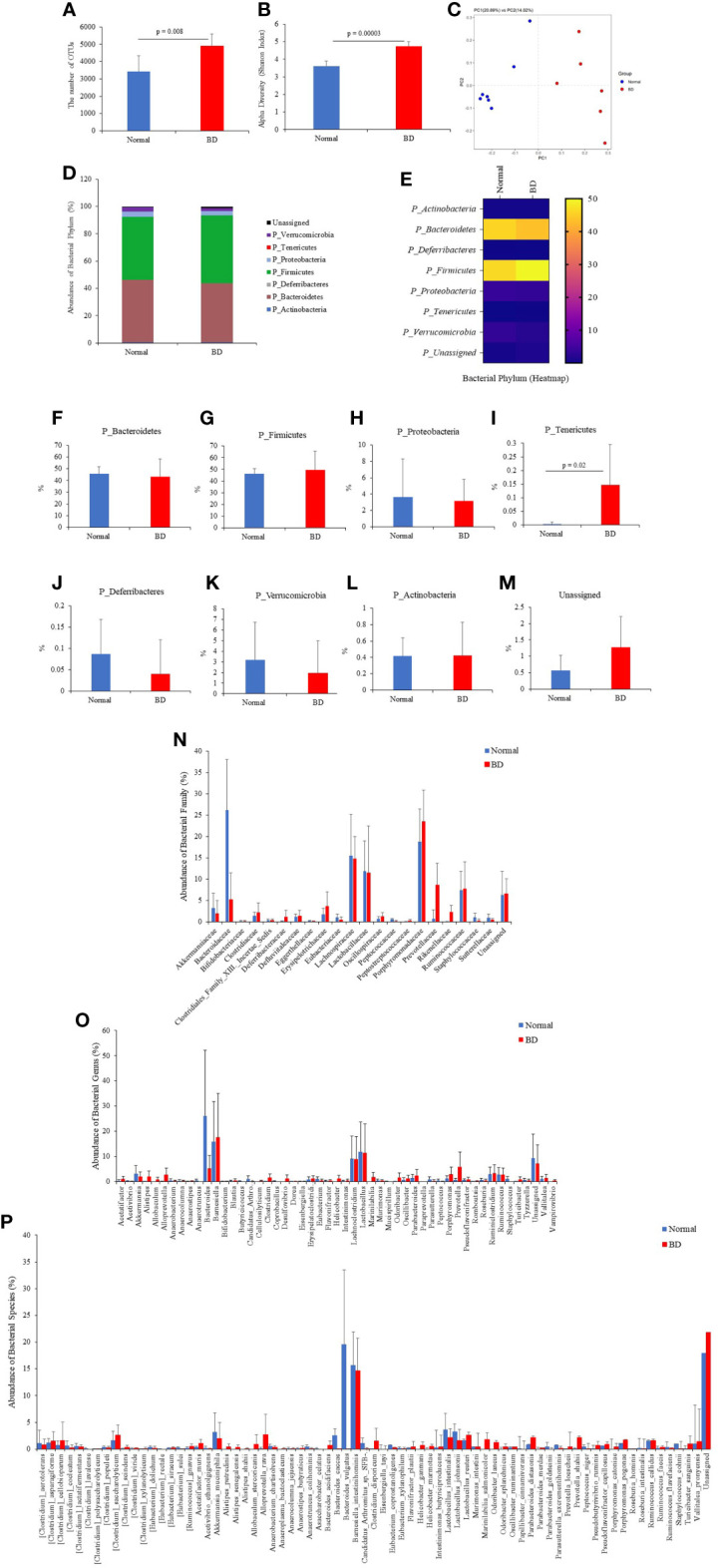
Fresh fecal samples were collected from normal (n = 7) and BD mice (n = 6), and metagenomic analysis was performed. Sequencing data from 16S rRNA V3 and V4 amplicons were used to perform microbiome analysis. BD mice 16S rRNA amplicon metagenomic analysis revealed an altered gut microbiome composition compared to normal mice. It showed the operational taxonomic units (OTUs) **(A)**, alpha diversity by the Shannon index **(B)**, and beta diversity by principal coordinate analysis (PCoA) **(C)**. The number of phyla **(D–M)**, families **(N)**, genera **(O)**, and species **(P)** showed significant differences in fecal 16S rRNA sequencing between the normal and BD mice. The p-value was determined by the Mann-Whitney U test.

To investigate the composition and function of the gut microbiome in BD mice, 16S rRNA metagenomic analysis was performed in fecal samples from normal mice and BD mice. Based on a National Center for Biotechnology Information (NCBI) classifier comparison of the microbial community, the taxonomic construct distances between the normal and BD mice were determined using the Shannon diversity index and beta diversity and visualized by a principal coordinate analysis (PCoA) plot (**Figures 4A–C**). According to the fecal sample microbiome analysis, the number of OTUs (p = 0.008) and alpha diversity (Shannon index, p = 0.00003) were significantly increased in BD mice compared to the normal mice, indicating that the BD mice had lower microbial diversity (**Figures 4A, B**). **Figure 4D** shows the abundance of bacterial flora and **Figure 4E** is the heat map of the bacterial flora. The results showed that *Bacteroidetes, Firmicutes*, *and Proteobacteria* were the dominant phyla in both the normal and BD mice (**Figures 4F–H**). Phylum *Tenericutes* (p = 0.02) was significantly higher in BD mice than in normal mice (**Figure 4I**). The expression of *Deferribacteres* and *Verrucomicrobia* was lower in BD mice than in normal mice, but *Actinobacteria* did not differ between the two groups, and the unassigned bacterial phylum was higher in BD mice (**Figures 4J–M**). **Supplementary Table ST2** shows the differences in fecal microbiota between the normal and BD mice and the *p*-values.

Among the microbial families, *Bacteroidaceae* (p = 0.002), *Eggerthellaceae* (p = 0.01), and *Peptococcaceae* (p = 0.0009) were significantly lower in BD mice than in normal mice (**Figure 4N** and **Supplementary Table ST2**). *Prevotellaceae* (p = 0.003) and *Rikenellaceae* (p = 0.002) were significantly higher in BD mice than in normal mice. However, the most dominant microbial families *Lachnospiraceae*, *Lactobacillaceae*, and *Porphyromonadaceae* were not significantly different between the groups. At the genus level, *Anaerotruncus* (p = 0.005), *Bacteroides* (p = 0.002), *Butyricicoccus* (p = 0.008), *Coprobacillus* (p = 0.02), *Mucispirillum* (p = 0.02), *Peptococcus* (p = 0.008), and *Staphylococcus* (p = 0.02) were significantly lower in BD mice than in normal mice (**Figure 4O**, **Supplementary Table ST2**). In contrast, *Acetatifactor* (p = 0.05), *Alistipes* (p = 0.004), *Desulfovibrio* (p = 0.01), *Helicobacter* (p = 0.04), *Odoribacter* (p = 0.007), *Prevotella* (p = 0.006), and *Vampirovibrio* (p = 0.006) were significantly higher in BD mice than in normal mice (**Figure 4O** and **Supplementary Table ST2**).

In the bacterial species, Clostridium lavalense (p = 0.03), Eubacterium sulci (p = 0.002), Anaerotruncus colihominis (p = 0.02), Asaccharobacter celatus (p = 0.01), Bacteroides caccae (p = 0.003), Bacteroides vulgatus (p = 0.005), Eubacterium xylanophilum (p = 0.02), Parabacteroides goldsteinii (p = 0.01), Peptococcus niger (p = 0.008), Roseburia intestinalis (p = 0.005), and Staphylococcus cohnii (p = 0.03) were significantly lower in the BD mice compared to the normal mice (**Figure 4P** and **Supplementary Table ST2**), whereas Clostridium scindens (p = 0.03), Acetatifactor muris (p = 0.05), Alistipes putredinis (p = 0.01), Alistipes senegalensis (p = 0.05), Anaeroplasma bactoclasticum (p = 0.03), Bacteroides acidifaciens (p = 0.04), Helicobacter ganmani (p = 0.08), Helicobacter marmotae (p = 0.03), Odoribacter laneus (p = 0.01), Odoribacter splanchnicus (p = 0.005), Parabacteroides merdae (p = 0.04), Prevotella loescheii (p = 0.01), and Porphyromonas catoniae (p = 0.01) were significantly higher in the BD mice than the normal mice (**Figure 4P** and **Supplementary Table ST2**).

### Butyrate Treatment Increases the Frequency of CD86+ Cells and Improves Symptoms in BD Mice

**Figure 5:**
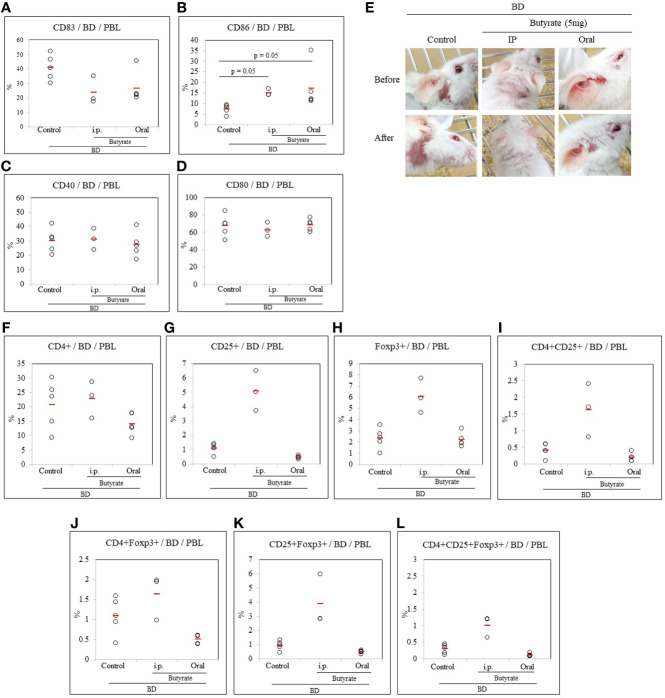
Butyrate affected the frequency of CD83+ cells in BD mice. In BD mice treated with butyrate, the frequency of DC co-stimulatory molecules CD83, CD86, CD40, and CD80 was evaluated by flow cytometric analysis of PBL surface staining **(A–D)**. The intraperitoneal or oral administration of butyrate to BD mice decreased the frequency of CD83+ cells **(B)** and significantly increased the frequency of CD86+ cells **(D)**. Butyrate treatment improved the symptoms in BD mice **(E)**. The frequency of regulatory T cells was analyzed by FACS after the intraperitoneal or oral administration of butyrate to BD mice **(F–L)**. The p-value was determined by the Kruskal-Wallis test. The experiments were performed independently at least three times.

Butyrate was administered intraperitoneally and orally to BD mice. The frequency of DCs expressing co-stimulatory molecules CD83, CD86, CD40, and CD80 in the PBL of butyrate-treated BD mice was measured by FACS analysis. The frequency of CD83+ cells was downregulated in butyrate intraperitoneally-treated BD mice compared to untreated BD mice (23.83 ± 9.32% *vs.* 40.72 ± 8.91%) but the difference was not statistically significant (**Figure 5A**). CD86+ cells were significantly increased in BD mice administered butyrate intraperitoneally (14.97 ± 1.76% *vs.* 7.28 ± 2.18%, *p* = 0.05) and orally (17.04 ± 10.24% *vs*. 7.28 ± 2.18%, *p* = 0.05) compared to untreated BD mice (**Figure 5B**). No statistical difference was observed in the frequency of CD40+ cells and CD80+ cells after butyrate treatment (**Figures 5C, D**). Butyrate treatment of BD mice showed improvement in symptoms (**Figure 5E**).

### Butyrate Treatment Increases Regulatory T Cells in BD Mice

Butyrate was administrated intraperitoneally and orally to BD mice to determine whether butyrate could modulate Treg cells. Treg cell markers CD4, CD25, and Foxp3 were analyzed in PBL by FACS analysis. Intraperitoneal injection or oral administration of butyrate to BD mice couldn’t make any significant difference in the frequency of CD4+ cells (**Figure 5F** **),** but intraperitoneal injection of butyrate to BD mice increased the frequency of CD25+ cells (5.07 ± 1.40% *vs.*1.08 ± 0.34%) and Foxp3+ cells (6.07 ± 1.56% *vs.*2.32 ± 0.92%) compared to untreated BD mice (**Figures 5G, H**) but the results were not significantly different. Intraperitoneal injection of butyrate to BD mice also increased the frequency of CD4+CD25+ cells (1.63 ± 0.80% *vs.*0.42 ± 0.20%) (**Figure 5I**), however no differences was observed in CD4+Foxp3+ cells (**Figure 5J**) among the groups. Intraperitoneal injection of butyrate to BD mice also increased the frequency of CD25+Foxp3+ cells (1.63 ± 0.57% *vs.* 1.09 ± 0.45%), and CD4+CD25+Foxp3+ cells (1.01 ± 0.32% *vs.* 0.29 ± 0.13%) compared to untreated BD mice (**Figures 5K, L**) but no statistically significant differences were observed. No difference was observed in the frequency of CD4+, CD8+, CD11b+, and CD11c+ cells in the LN cells of BD mice treated intraperitoneally or orally with butyrate (**Supplementary Figure S1**).

### Dose Dependence of *Eubacterium rectale* on Dendritic Cell Activation Marker Expression in Normal and BD Mice

**Figure 6:**
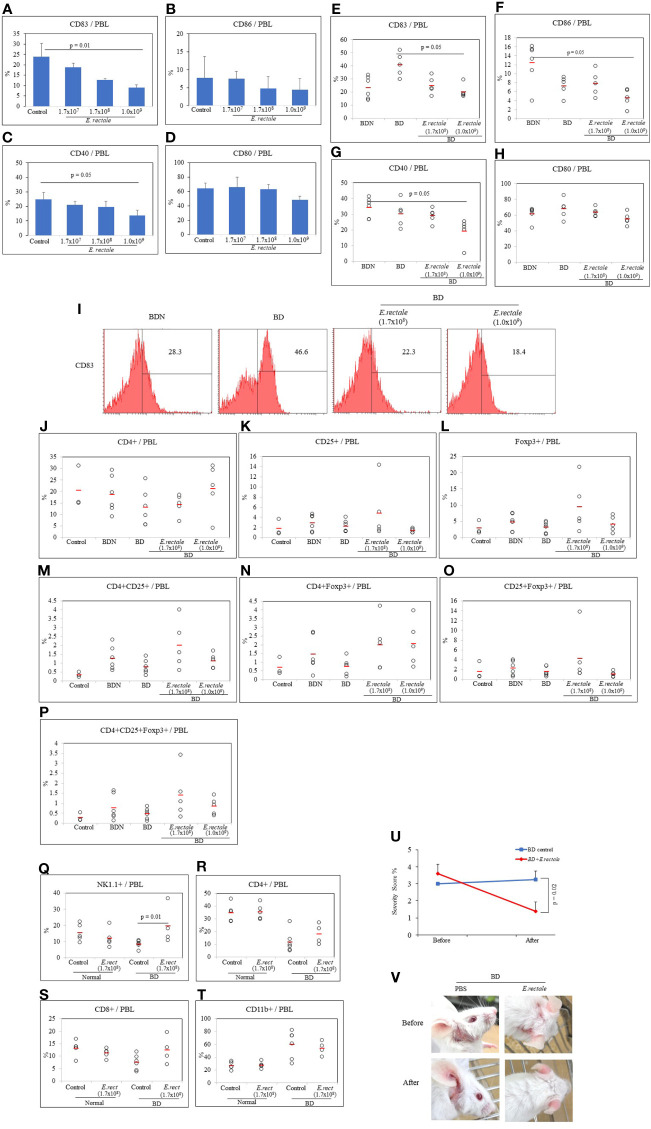
*Eubacterium rectale* administration regulated dendritic cell activation and regulatory T cells in normal mice. The frequencies of CD83, CD86, CD40 and CD80 on the PBL surface of normal mice treated with *E. rectale* (1.7x107, 1.7x108, 1.0x109 CFU) for 10 consecutive days were evaluated by flow cytometry **(A–D)**. The frequencies of CD83, CD86, CD40 and CD80 on the surface of the PBL of BD mice treated with *E. rectale* (1.7x108 and 1.0x109 C.F.U) were assessed by flow cytometry **(E–H)**. Representative histograms of the frequency of CD83+ in PBL of BD mice **(I)**. The frequency of regulatory T cells was analyzed by FACS in normal control, BDN, BD and BD mice treated with *E. rectale* **(J–P)**. The frequencies of NK1.1+, CD4+, CD8+ and CD11b+ cells in normal, *E. rectale*-treated normal, BD and *E. rectale*-treated BD mice were evaluated by FACS analysis **(Q–T)**. Administration of *E. rectale* (1.7x108) for 10 consecutive days significantly reduced the severity score **(U)** and improved symptoms **(V)** in BD mice. Experiments were performed independently at least three times. The p-value was determined by the Kruskal-Wallis test.

Probiotics must be administered in adequate amounts to achieve the benefits. As a result of intraperitoneal and oral administrations of butyrate, Treg expression was different. Therefore, a dose-dependence on direct bacterial administration was required. The dose-response effect was tested to determine the appropriate amount of *E. rectale* to administer to BD mice. After oral administration of *E. rectale* to normal mice, the frequency of dendritic cell activation marker-positive cells was analyzed by FACS. The dendritic cell activation markers used were CD40, CD83, CD80, and CD86. Three doses of *E. rectale* (1.7x10^7^, 1.7x10^8^ and 1.0x10^9^ C.F.U/mouse) were administered orally to normal mice for 10 consecutive days once a day. *E. rectale* treatment at 1.0x10^9^ C.F.U significantly reduced the frequency of CD83+ cells compared to the controls (9.00 ± 1.29% *vs.* 23.97 ± 6.38%, p = 0.01) (**Figure 6A**). No significant difference was observed in CD86+ cell frequency after *E. rectale* treatment (**Figure 6B**). The frequency of CD40+ cells were also significantly downregulated in the 1.0x10^9^ C.F.U of *E. rectale-*treated group compared to the controls (13.65 ± 3.89% *vs.* 24.95 ± 4.73%, p = 0.05) (**Figures 6C**). No significant difference was observed in CD80+ cell frequency after *E. rectale* treatment (**Figure 6D**). Treatment of BD mice with 1.0x10^9^ C.F.U of *E. rectale* per mouse significantly reduced the frequency of CD83+ cells compared to untreated BD mice (20.20 ± 5.14% *vs.* 40.72 ± 8.91%, *p* = 0.05) (**Figure 6E**). The frequency of CD86+ cells and CD40+ cells was also reduced in *E. rectale* (1.0x10^9^ C.F.U/mice)-treated BD mice compared to BDN mice (4.60 ± 2.0% *vs.* 12.45 ± 4.62%, *p* = 0.05; 19.16 ± 3.88% *vs.* 34.27 ± 6.20%, *p* = 0.05, respectively) (**Figure 6F, G**). No significant difference was found in CD80+ cell frequency (**Figure 6H**). A representative histogram of CD83+ cell frequency in *E. rectale*-treated BD mice is shown in **Figure 6I**.

### *Eubacterium rectale* Treatment Modulates Regulatory T Cells in BD Mice

CD4+CD25+Foxp3+ Treg cells were analyzed by FACS in *E. rectale-*treated BD mice. The frequencies of CD4+ and CD25+ cells in *E. rectale-*treated BD mice were not significant among the groups (**Figures 6J, K**). The frequency of Foxp3+ cells and CD4+CD25+ cells was increased after *E. rectale* (1.7x10^8^ C.F.U/mouse) treatment compared to untreated BD mice (9.36 ± 7.94% *vs.* 3.12 ± 1.75; 2.0 ± 1.36% *vs.* 0.78 ± 0.40, respectively) (**Figures 6L, M**). The CD4+Foxp3+ cell frequency was also increased after *E. rectale* (1.0x10^9^ C.F.U/mouse) treatment compared to untreated BD mice (2.07 ± 1.30% *vs.* 0.75 ± 0.46), (**Figure 6N**), but the frequencies of CD25+Foxp3+ cells in *E. rectale-*treated BD mice were not significant among the groups (**Figure 6O**). The frequencies of CD4+CD25+Foxp3+ regulatory T cell was upregulated after *E. rectale* (1.7x10^8^ C.F.U/mouse) treatment, but the difference was not statistically significant (1.40 ± 1.21% *vs.* 0.25 ± 0.22) (**Figure 6P**).

### *Eubacterium rectale* Treatment Upregulates NK Cells in BD Mice

The frequency of NK1.1+ cells in BD mice was lower than in normal mice (8.30 ± 2.36% *vs.* 15.34 ± 5.40%) but the difference was not statistically significant. BD mice administered *E. rectale* upregulated NK1.1+ cells compared to non-administered BD mice (19.52 ± 11.79% *vs.* 8.30 ± 2.36%, p = 0.01) (**Figure 6Q**). The frequency of CD4+ cells and CD8+ T cells in the PBL of BD mice was lower than in normal mice (11.82 ± 8.54% *vs.* 34.78 ± 7.25%; 7.42 ± 3.06% *vs.* 13.10 ± 3.19%, respectively) (**Figures 6R, S**). In contrast, the frequency of CD11b+ cells were higher in BD mice than in normal mice (59.52 ± 21.37% *vs.* 26.82 ± 6.12%) (**Figure 6T**) but no statistically significant difference was observed between the groups.

### *Eubacterium rectale* Treatment Reduces Disease Severity Scores and Improves Symptoms in BD Mice

To determine whether *E. rectale* was able to manage BD symptoms, *E. rectale* was orally administered to BD mice for 10 consecutive days, and symptoms were tracked during that period. BD mice treated with *E. rectale* culture media were used as controls and maintained for the same period. The severity score of *E. rectale*-treated BD mice decreased significantly after 10 days of treatment compared to that of the control-treated BD mice (1.34 ± 0.54% *vs.* 3.25 ± 0.5%, *p* = 0.02) (**Figure 6U**). **Figure 6V** shows the changes in BD symptoms 10 days after the administration of *E. rectale.*

### Co-Administration of Colchicine and *Eubacterium rectale* Downregulates CD83 in BD Mice

**Figure 7:**
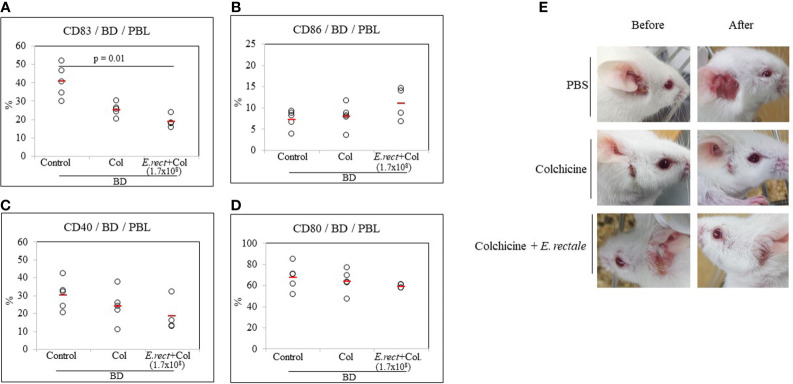
The frequency of DC activation markers in BD mice after the administration of colchicine alone or in combination with *E. rectale*. The frequency of CD83, CD86, CD40, and CD80 on the surface of PBL of BD mice was evaluated by FACS analysis **(A–D)**. Changes in symptoms after treatment **(E)**. The p-value was determined by the Kruskal-Wallis test.

Colchicine is a drug frequently used to treat patients with BD and has been reported to significantly reduce the symptoms (38, 39). Colchicine administration to BD mice reduced the frequency of CD83+ cells compared to untreated BD mice (25.28 ± 3.55% *vs* 40.72 ± 8.91%) (**Figure 7A**) but the difference was not statistically significant. The combined administration of colchicine and *E. rectale* to BD mice significantly reduced the frequency of CD83+ cells compared to untreated BD mice (18.67 ± 3.59% *vs.* 40.72 ± 8.91%, p = 0.01) (**Figure 7A**). The administration of colchicine alone or in combination with *E. rectale* did not show statistically significant differences in the frequency of CD86+, CD40+, and CD80+ cells between the groups (**Figures 7B–D**). Colchicine alone or in combination with *E. rectale* improved BD symptoms in mice (**Figure 7E**). After the administration of colchicine alone or in combination with *E. rectale* to BD mice, Treg cells were not upregulated in PBL (**Supplementary Figure S2**). The frequencies of CD4+, CD8+, CD11b+, and CD11c+ cells in the LN of BD mice treated with colchicine alone or in combination with *E. rectale* were not significant among the groups (**Supplementary Figure S3**).

### Lyophilized *Eubacterium rectale* Reduces the Frequency of CD83+ Cells in BD Mice

**Figure 8:**
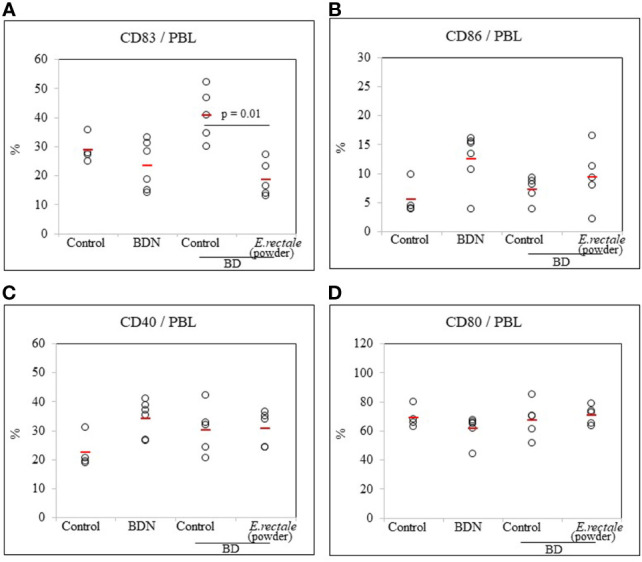
FACS analysis of the frequency of DC activation markers in BD mice after administration of lyophilized *E. rectale* **(A–D)**. The p-value was determined by the Kruskal-Wallis test. The experiments were performed more than three independent times.

Whether *E. rectale* in the form of freeze-dried powder could regulate CD83+ cells in BD mice was investigated. As a result of the oral administration of 0.5 mg of freeze-dried *E. rectale* to BD mice (once daily, 10 consecutive days), the frequency of CD83+ cells in BD mice was significantly reduced compared to untreated BD mice (18.62 ± 6.17% *vs*. 40.72 ± 8.91%, p = 0.01) (**Figure 8A**). No significant differences were observed in the frequency of CD86+, CD40+, or CD80+ cells after freeze-dried *E. rectale* treatment (**Figures 8B–D**). Freeze-dried *E. rectale* treatment had no effect on the increase in Treg cell frequencies compared to control (**Supplementary Figure S4**).

### *Eubacterium rectale* Reduces Serum IL-17 Levels in BD Mice

**Figure 9:**
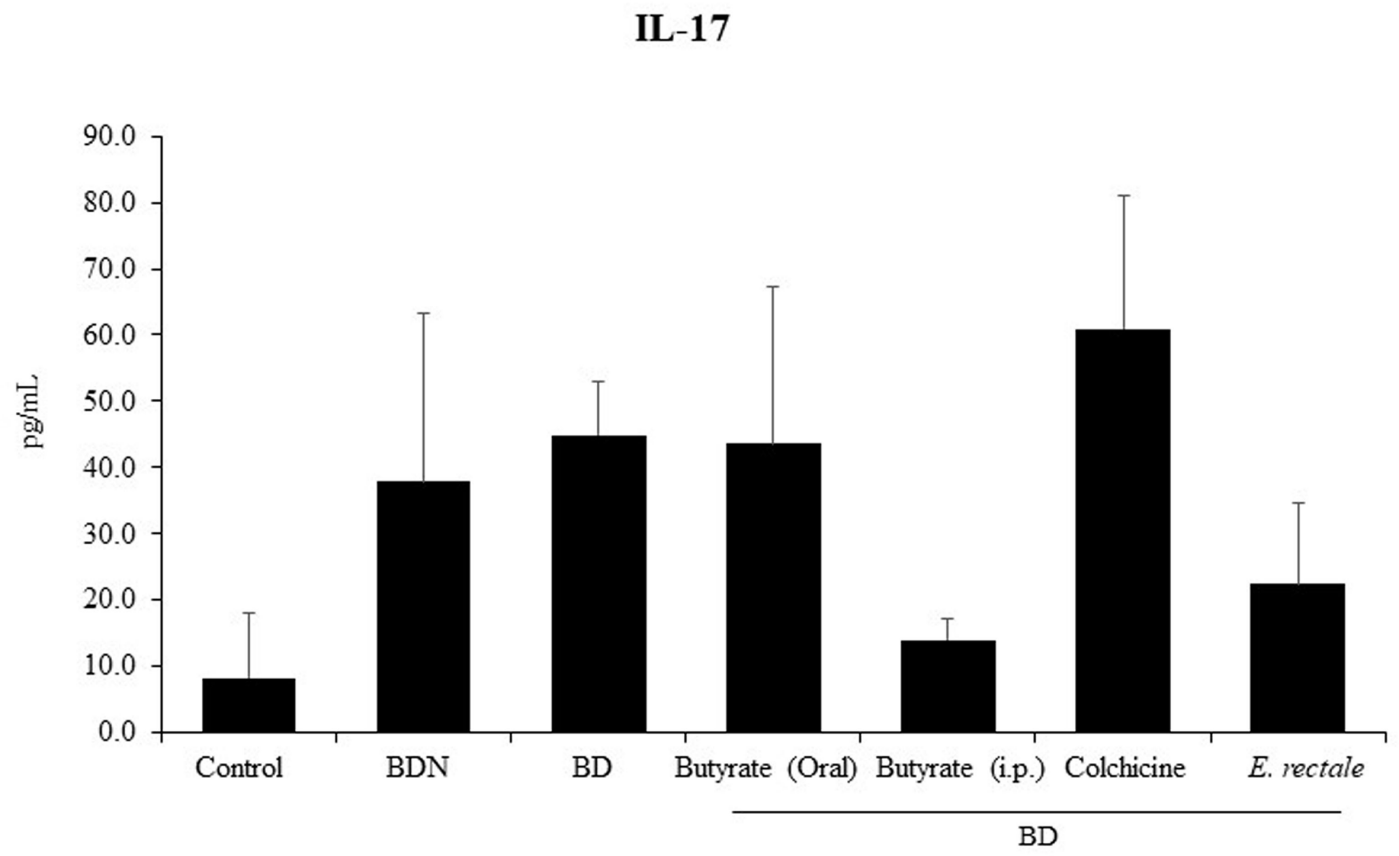
Serum IL-17 levels in BD mice treated with butyrate orally or intraperitoneally, *E. rectale*, or colchicine were analyzed by ELISA. The p-value was determined by the Kruskal-Wallis test.

Several studies on BD have demonstrated that a significant increase in serum IL-17 is an indicator of relapse or infection recurrence (40, 41). To determine whether *E. rectale* administration could improve BD symptoms by downregulating IL-17, serum IL-17 levels were measured by ELISA in BD mice administered *E. rectale*. BD mice showed higher concentrations of IL-17 than control mice (45.0 ± 8.05 pg/mL *vs.* 8.30 ± 9.77 pg/mL), statistically it was not significant. Intraperitoneal treatment with butyrate downregulated serum IL-17 levels in BD mice compared to the untreated BD mice (22.05 ± 12.30 pg/mL *vs.* 45.0 ± 8.05 pg/mL). Serum IL-17 levels were downregulated in *E. rectale-*treated BD mice compared to untreated BD mice (22.05 ± 12.30 pg/mL *vs.* 45.0 ± 8.05 pg/mL) (**Figure 9**). However, no statistical significance was observed between the groups.

## Discussion

Recently, research on microbiota has been actively conducted. Dysbiosis, an imbalance in gut microflora, can lead to many metabolic and inflammatory pathologies, such as BD (12, 27, 42), multiple sclerosis (43), RA (44), ankylosing spondylitis (45), and inflammatory bowel disease (46). BD patients showed reduced gut microbial diversity (47). Fecal bacterial transplantation from BD patients was found to significantly exacerbate uveitis in B10RIII mice (12). This suggests that controlling the microflora may also have the potential to improve BD symptoms. Patients with active BD have low levels of butyrate-producing bacteria (12). Bacterial species exert anti-inflammatory effects by secreting several short-chain fatty acids, including butyrate, one of the major metabolites of the gut microflora (48). *In vitro* human monocyte-derived DCs treated with butyrate revealed a strong downregulation of CD83 (49). After confirming the association between BD inflammation and CD83, and the association between BD inflammation and gut microbiota, whether inflammation could be alleviated by regulating intestinal bacteria was investigated. In particular, *E. rectale* was selected as a butyrate-secreting strain because it has the advantage of inhabiting the human colon. *Eubacterium* was also one of the most prevalent commensals in healthy populations compared to COVID-19 patients (50).

In BD patients with ocular symptoms, *Bacteroidetes*, *Firmicutes*, and *Proteobacteria* were the dominant phyla in the gut microbiota (12). In our study, BD mice showed a similar tendency. The phylum *Tenericutes* was higher in BD mice. *Tenericutes* bacteria were also highly abundantly expressed in the pathological setting of patients with Crohn’s disease (51).

The families *Bacteroidaceae* and *Prevotellaceae* were significantly upregulated in BD mice. *Bacteroidaceae* and *Prevotellaceae* were decreased after IL-23 inhibition in SKG mice. IL-23 favors the outgrowth of spondyloarthritis-associated pathogens (52). IL-23 was the most frequently detectable cytokine in active Behçet’s patients (53). *Bacteroidaceae* and *Prevotellaceae* may affect BD inflammation through IL-23 production. The family *Peptococcaceae* was found to be significantly downregulated in BD mice compared to normal healthy mice. *Peptococcaceae* bacteria were also downregulated in mice with a disrupted gut barrier induced by cyclophosphamide (54). *Peptococcaceae* may play a protective role in BD intestinal symptoms.

The genus *Prevotella* was significantly higher in BD patients (12) and BD mice. *Prevotella* expansion was also observed in the ileum of intestinal inflamed mice (55). *Prevotella* strain *P. intermedia* was one of the periopathogens with an association between periodontal pathogenicity and atheromatous plaques (56). *Prevotella* appears to contribute to the generation of inflammation in various diseases.

The genus *Bacteroides* was downregulated in BD patients (57) and BD mice compared to normal controls. *Bacteroides* strains have good and bad features (58). Strains *B. caccae* and *B. vulgatus*, showing good properties, were downregulated in BD mice. *B. caccae* plays a role in pectin fermentation and secretes acetate (59). Acetate is known to attenuate inflammasome activation (60). Increased inflammasome expression has been reported in BD patients with skin manifestations (61) and BD patients with intestinal problems (62). *B. vulgatus* attenuated atherosclerosis through *Foxp3* upregulation and TNFα downregulation (63). *Foxp3* has been shown to be a master regulator of Treg cell function (64), and the upregulation of Treg cells ameliorated inflammatory BD symptoms (65). TNFα downregulation correlated with an improvement in BD symptoms in mice (66) and patients (67).

The genus *Butyricicoccus* was downregulated in BD mice and models of inflammatory bowel disease. *Butyricicoccus* strain *B. pullicaecorum* was able to attenuate chemically induced colitis through the downregulation of TNFα (68).

CD83 is a DC activation marker and is also expressed on monocytes, B cells, NK cells, and T cells (24). DCs are a key initiator and are involved in innate and adaptive immunity at the forefront of the immune response (69). In an inflammatory environment, autoreactive T cells are initially activated by DCs and activated T cells secrete TNF and IL-17, which recruit macrophages and other inflammatory cytokines (18, 70). Activated DCs in humans and mice highly and stably express CD83 (25). The inhibition of CD83 improved BD symptoms in mice (26). This suggests that CD83 may play an important role in regulating the inflammatory response in BD. In this study, it was found that the frequency of CD83+ cells was higher in BD patients with arthritis than in healthy controls. Consistent with expression in the patients, there was also a higher frequency of CD83+ cells in BD mice. Therefore, CD83 inhibition through regulation of the gut microbiota could be a target for inflammation management. Butyrate treatment downregulated CD83+ cell frequency and upregulated CD86+ cell frequency. Abatacept, a modulator of the CD80/CD86-CD28 signal for T cell activation, also downregulated CD83+ cell frequency and upregulated CD86+ cell frequency, with a trend similar to that of butyrate. Butyrate and Abatacept treatment-induced improvement did not affect the Treg cell population (26), whereas, *E. rectale* treatment downregulated only CD83+ cells, not CD86+ cells. According to P. Yang, CD86 expression decreased after treatment with 5-Aza-2’-deoxycytidine, a DNA demethylating drug, for ocular symptoms in BD patients. CD83 expression was not altered by this treatment (71).

Colchicine is an effective medicine frequently prescribed for BD patients (72, 73). Colchicine alone or in combination with rebamipide improved BD symptoms in mice (2, 74). In this study, the combination of colchicine and *E. rectale* significantly reduced the frequency of CD83+ cells compared to colchicine alone. Colchicine treatment was not able to significantly increase the proportion of Foxp3+ Treg cells in BD patients (75). Similarly, colchicine alone or in combination with *E. rectale* did not modulate the frequency of Treg cells in BD mice. Taken together, it was confirmed that BD symptoms could be improved by regulating the expression of DC activation-related markers. BD has widespread symptoms and affects many organs, including the mouth, skin, genitals, eyes, intestines, and joints (76). More research is needed on whether antigen-presenting cell activation markers change differently depending upon the symptoms, composition of the symptoms, and the treatment of BD.

Lyophilization is a process for improving the stability of a product in the form of a dry powder without losing the characteristics of the product. The process involves removing moisture from the product in a vacuum at a sufficiently low temperature (77, 78). Freeze-dried storage of bacterial strains provides ease of use. The oral administration of *E. rectale* lyophilized powder showed a similar downregulation of CD83, and this form can be applied to human applications.

In this study, we found that increased levels of CD83+ cells were associated with disease symptoms in both BD patients and BD mice. Changes in the composition of the intestinal microbiota using the colon-resident bacterial strain *E. rectale* showed a therapeutic effect in BD mice, accompanied by modulation of the DC activation marker phenotype. Thus, *E. rectale* is expected to be applied to patients with systemic inflammatory BD in the future.

## Data Availability Statement

The16S rRNA metagenomic data has been deposited in NCBI under the accession number PRJNA723404.

## Ethics Statement

The studies involving human participants were reviewed and approved by Institutional Review Board of Ajou University Hospital (approval number: AJIRB-BMR-SMP-13-398). The patients/participants provided their written informed consent to participate in this study. The animal study was reviewed and approved by Institutional Animal Care and Use Committee of Ajou University (approval number: AMC-2018-0017).

## Author Contributions

SS contributed to the concept and design of the study as well as data analysis and interpretation. SMSI, HOB, and HMR participated in data acquisition, analysis, and interpretation. SI and HS cared for the animals. JYJ, HAK, and CHS provided human samples. SS and SMSI wrote the manuscript. All authors contributed to the article and approved the submitted version.

## Funding

This research was supported by a grant (2017R1D1A1B03032168) from the Basic Science Research Program through the National Research Foundation of Korea (NRF) funded by the Ministry of Education, Science, and Technology. It was also supported by an NRF grant funded by the Korean government (MIST) (2020R1A2C2012721) and the Korea Health Technology R&D Project through the Korea Health Industry Development Institute, funded by the Ministry of Health & Welfare, Republic of Korea (HI16C0992).

## Conflict of Interest

The authors declare that the research was conducted in the absence of any commercial or financial relationships that could be construed as a potential conflict of interest.

## Publisher’s Note

All claims expressed in this article are solely those of the authors and do not necessarily represent those of their affiliated organizations, or those of the publisher, the editors and the reviewers. Any product that may be evaluated in this article, or claim that may be made by its manufacturer, is not guaranteed or endorsed by the publisher.

## Supplementary Material

Supplementary Figure 1 The frequency of CD4+, CD8+, CD11b+, and CD11c+ cells in lymph node (LN) cells of BD mice treated orally and intraperitoneally with butyrate was assessed by FACS analysis **(A–D)**. The p-value was determined by the Kruskal-Wallis test. The experiments were performed independently at least three times.

Supplementary Figure 2 The frequency of regulatory T cells in BD mice treated with colchicine or the combination of colchicine and *E. rectale* was analyzed by FACS **(A–G)**. Untreated BD mice were used as the control group. The p-value was determined by the Kruskal-Wallis test. The experiments were performed independently at least three times.

Supplementary Figure 3 The frequency of CD4+, CD8+, CD11b+, and CD11c+ cells in LN of BD mice treated with colchicine or the combination of colchicine and *E. rectale* was analyzed by FACS **(A–D)**. Untreated BD mice were used as the control group. The p-value was determined by the Kruskal-Wallis test. The experiments were performed independently at least three times.

Supplementary Figure 4 The frequency of regulatory T cells in BD mice treated with *E. rectale* in culture solution or lyophilized *E. rectale* powder was analyzed by FACS **(A–G)**. The p-value was determined by the Kruskal-Wallis test. The experiments were performed independently at least three times.
