# Supplementary material for: Eubacterium rectale Attenuates HSV-1 Induced Systemic Inflammation in Mice by Inhibiting CD83
Source: Front Immunol. 2021 Aug 31;12:712312. doi: 10.3389/fimmu.2021.712312 (PMC8438521; doi:10.3389/fimmu.2021.712312)

Supplementary Figure S1

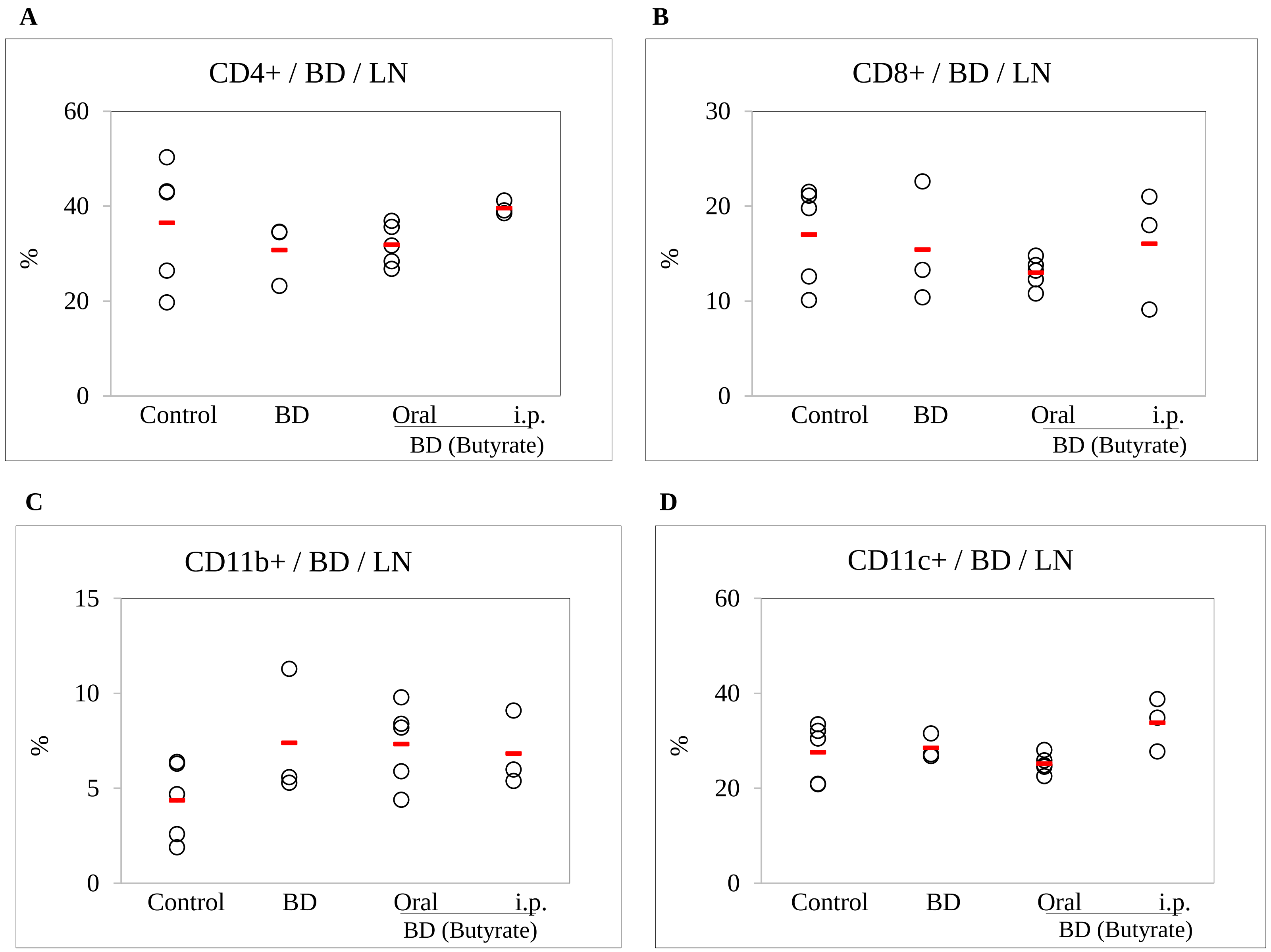

Supplementary Figure S2

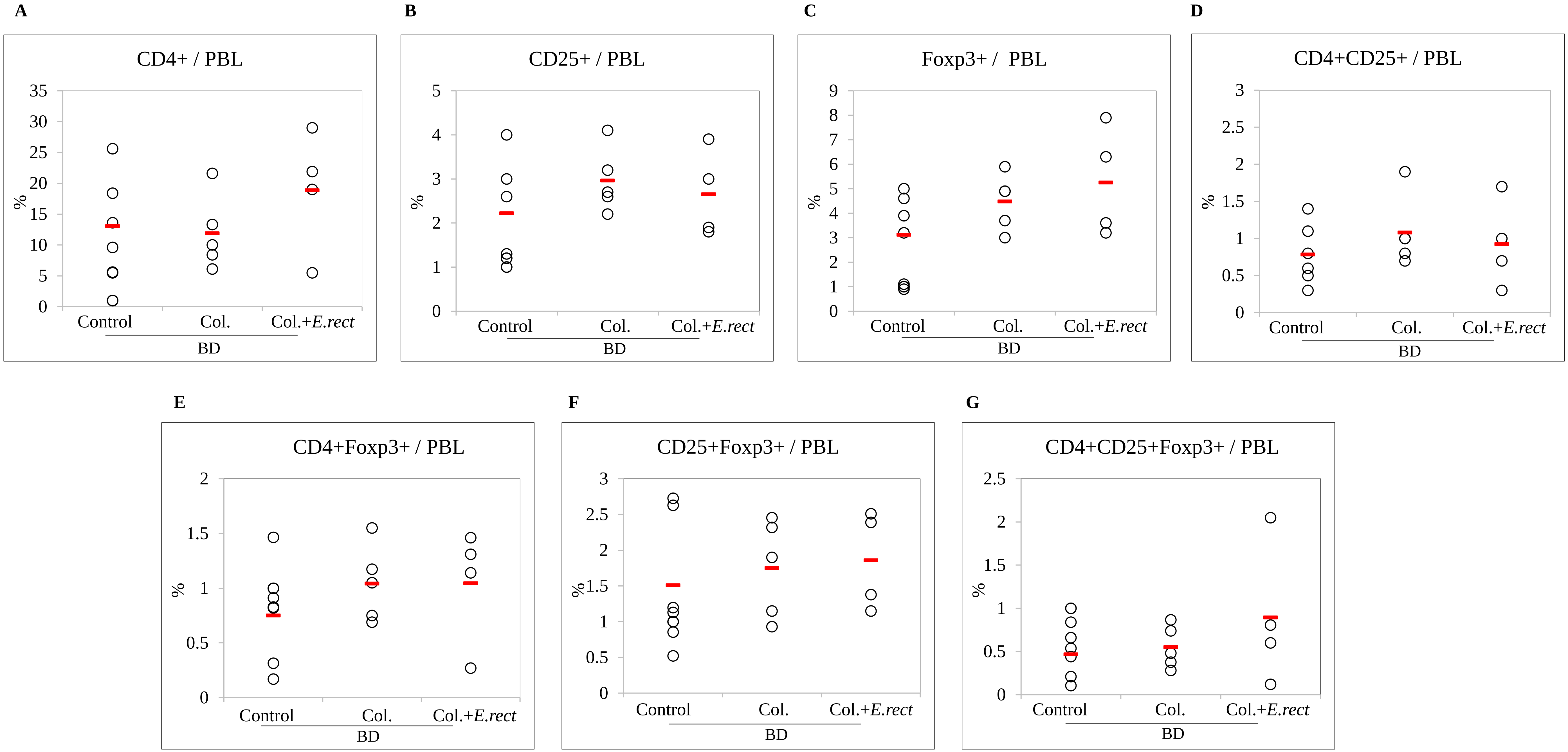

Supplementary Figure S3

A

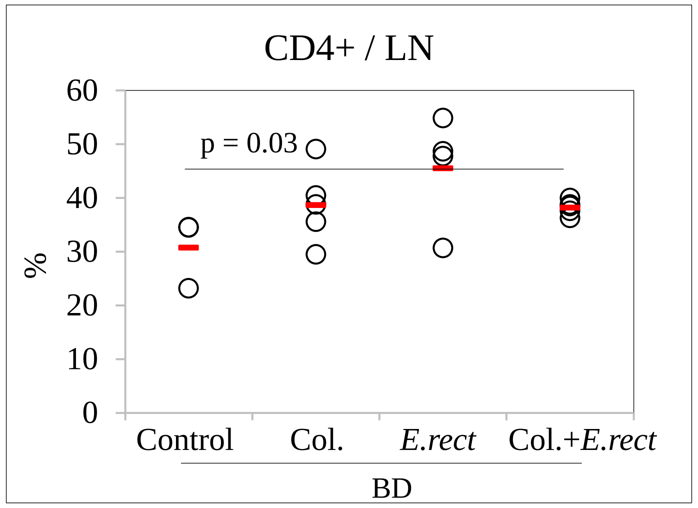

B

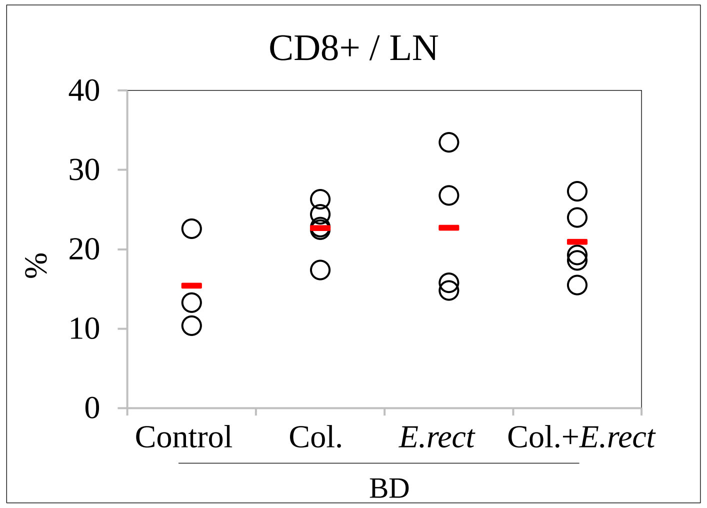

C

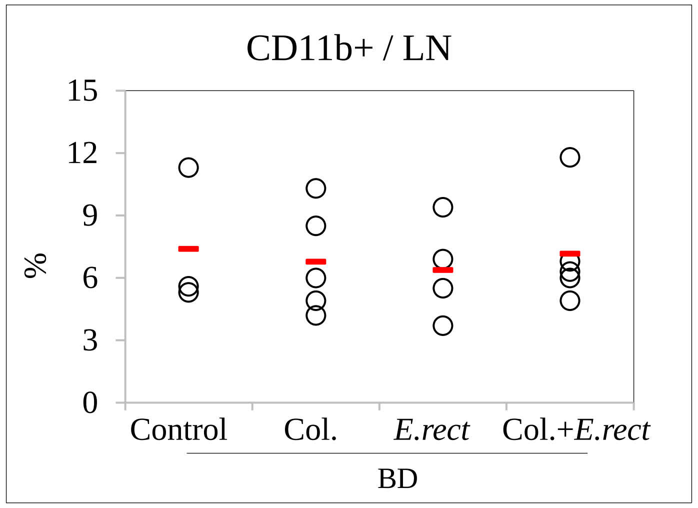

D

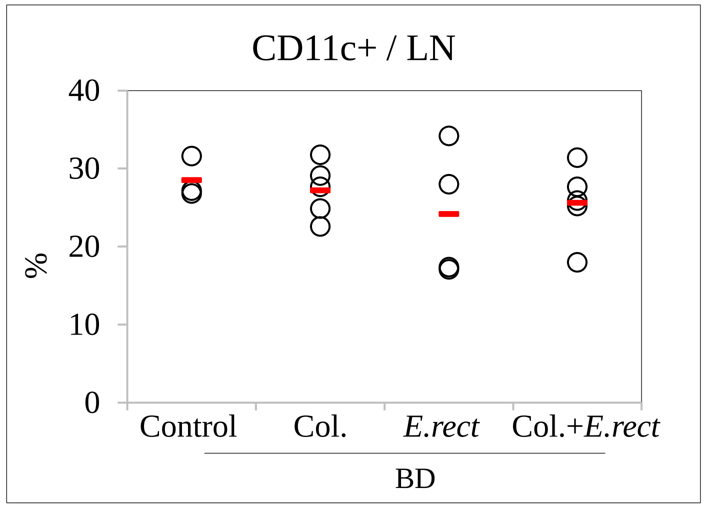

Supplementary Figure S4

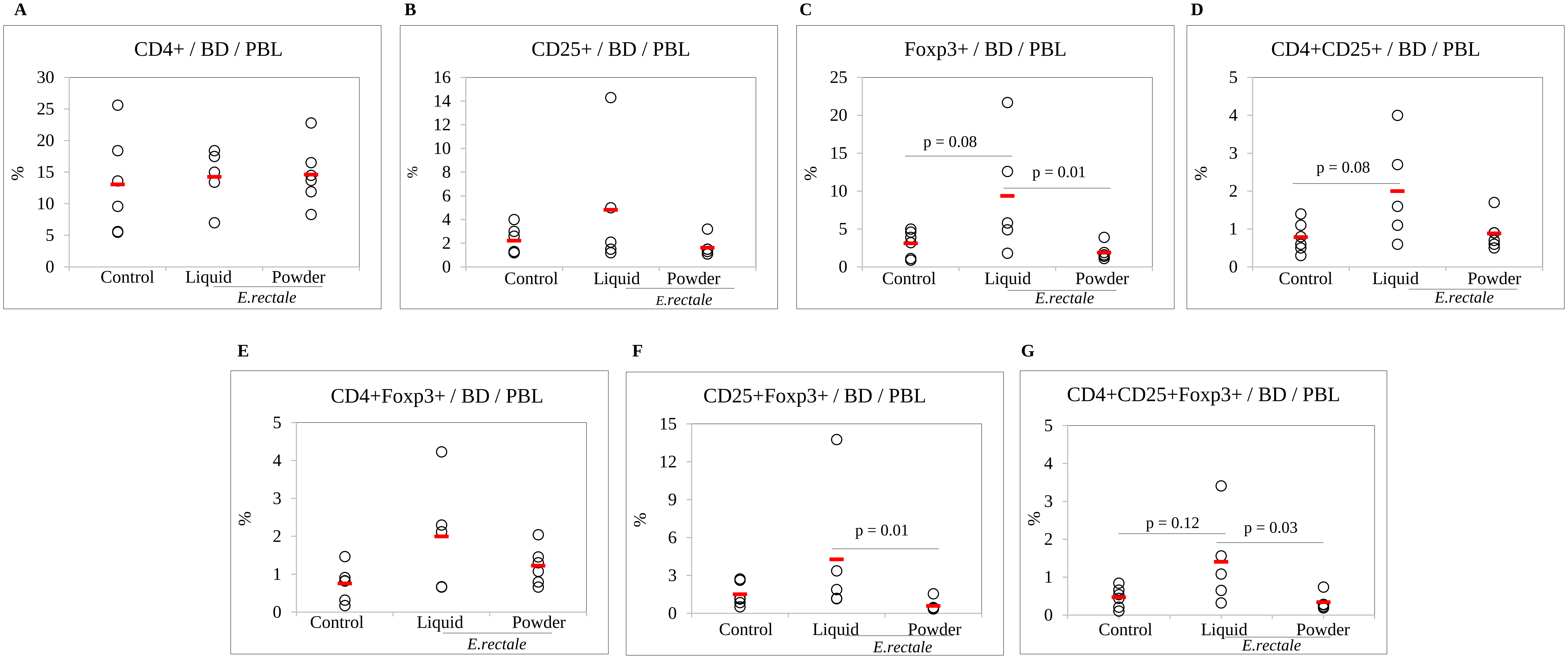

Supplement: Supplementary Figure 1 — The frequency of CD4+, CD8+, CD11b+, and CD11c+ cells in lymph node (LN) cells of BD mice treated orally and intraperitoneally with butyrate was assessed by FACS analysis (A–D). The p-value was determined by the Kruskal-Wallis test. The experiments were performed independently at least three times. [file DataSheet_1.pdf]
