# Supplementary material for: Eubacterium rectale Attenuates HSV-1 Induced Systemic Inflammation in Mice by Inhibiting CD83
Source: Front Immunol. 2021 Aug 31;12:712312. doi: 10.3389/fimmu.2021.712312 (PMC8438521; doi:10.3389/fimmu.2021.712312)
Supplement: Supplementary file 2 [file Table_1.docx]

**Supplementary Table ST1:** Sources of antibodies used in humans and mice in this work

| FACS markers | Fluorochromes | Company |
| --- | --- | --- |
| Anti-human antibodies | | |
| CD40 | FITC | eBioscience, San Diego, CA, USA |
| CD83 | APC | eBioscience, San Diego, CA, USA |
| CD80 | PE | eBioscience, San Diego, CA, USA |
| CD86 | Percp-eFluor 710 | eBioscience, San Diego, CA, USA |
| Anti-mouse antibodies | | |
| CD40 | PerCP-eFluro 710 | eBioscience, San Diego, CA, USA |
| CD83 | eFluro 660 | eBioscience, San Diego, CA, USA |
| CD80 | PE-Cyanine7 | eBioscience, San Diego, CA, USA |
| CD86 | FITC | eBioscience, San Diego, CA, USA |
| CD4 | FITC | eBioscience, San Diego, CA, USA |
| CD8 | PerCP-Cyanine 5.5 | Invitrogen, California, USA |
| CD11c | PE-Cyanine7 | eBioscience, San Diego, CA, USA |
| CD11b | PE | Invitrogen, California, USA |
| NK1.1 | PE-Cyanine7 | eBioscience, San Diego, CA, USA |
| Mouse Regulatory T cell staining markers | | |
| CD4 | PE-Cyanine7 | eBioscience, San Diego, CA, USA |
| CD25 | PE | eBioscience, San Diego, CA, USA |
| Foxp3 | PE-Cyanine5 | eBioscience, San Diego, CA, USA |
